# Expanding Repertoire of Plant Positive-Strand RNA Virus Proteases

**DOI:** 10.3390/v11010066

**Published:** 2019-01-15

**Authors:** Krin S. Mann, Hélène Sanfaçon

**Affiliations:** Summerland Research and Development Centre, Agriculture and Agri-Food Canada, Summerland, BC V0H 1Z0, Canada; mann.krin@gmail.com

**Keywords:** proteolytic processing, viral proteases, protease specificity, protease structure, virus evolution

## Abstract

Many plant viruses express their proteins through a polyprotein strategy, requiring the acquisition of protease domains to regulate the release of functional mature proteins and/or intermediate polyproteins. Positive-strand RNA viruses constitute the vast majority of plant viruses and they are diverse in their genomic organization and protein expression strategies. Until recently, proteases encoded by positive-strand RNA viruses were described as belonging to two categories: (1) chymotrypsin-like cysteine and serine proteases and (2) papain-like cysteine protease. However, the functional characterization of plant virus cysteine and serine proteases has highlighted their diversity in terms of biological activities, cleavage site specificities, regulatory mechanisms, and three-dimensional structures. The recent discovery of a plant picorna-like virus glutamic protease with possible structural similarities with fungal and bacterial glutamic proteases also revealed new unexpected sources of protease domains. We discuss the variety of plant positive-strand RNA virus protease domains. We also highlight possible evolution scenarios of these viral proteases, including evidence for the exchange of protease domains amongst unrelated viruses.

## 1. Introduction

Eukaryotic RNA viruses have a long evolution history, which is driven by their necessary adaptation to their hosts [1]. Viruses have likely evolved from capsid-less genetic parasites that later acquired various types of capsid proteins [2,3]. Thus, many viral replication proteins, notably RNA-dependent RNA polymerases (RdRps), helicases, and some proteases, have ancient origins, traced back to eukaryogenesis or even earlier [1,4]. Virus evolution has been described as modular [5]. Indeed, phylogenetic analyses of hallmark genes (capsid, RdRp, helicase) have revealed multiple examples of protein domain exchanges between viruses of different types. In addition, horizontal virus transfer between lower eukaryotes, invertebrates, plants, and vertebrates have also contributed to the evolution of eukaryotic viruses [6]. Plant viruses exemplify the modular evolution of RNA viruses. Progressive protein domain acquisitions by plant viruses can be partly attributed to the mixed infections that are typically observed in plants [7,8]. Most plant viruses depend on insect, nematode, or fungal vectors for plant-to-plant transmission, and in some cases, also replicate in these vectors [9,10,11]. Thus, selection pressure from both plant and insect hosts along with the heterogeneous population structure of RNA viruses have likely played a major role in the gain and/or loss of protein domains [11,12,13].

Positive-strand [(+)-strand] RNA viruses have a constrained genome size and have adapted to this limitation by encoding multifunctional proteins and/or by expressing different forms of their proteins using sophisticated expression strategies [14,15]. One strategy that is employed by many (+)-strand RNA viruses is to express large polyproteins that are subsequently processed into smaller functional gene products by viral and/or cellular proteases [16,17,18,19,20,21]. Polyprotein processing allows for the controlled and timely release of mature functional gene products or partially processed intermediate polypeptides that can differ in their biological activities. Thus, viruses have also developed various mechanisms to regulate the activity of viral proteases and/or the efficiency of cleavage of polyproteins at specific sites.

Positive-strand RNA viruses have been divided into two large groups, alpha-like or picorna-like based on the phylogeny of their RdRps [1]. Picorna-like viruses typically encode a main protease that cleaves at multiple sites in the large polyproteins [22,23]. They may also encode additional accessory proteases. In contrast, alpha-like viruses do not always encode a protease, and when they do, they are often leader proteases, auto-catalytically cleaving the polyprotein at a single site to promote their own release from the polyprotein [24,25].

Viral proteases are often multi-functional, playing key roles in multiple steps of the infection cycle. Indeed, they have been described to counteract antiviral defense responses by suppressing RNA silencing or having deubiquitinase activity, facilitate symplastic and systemic long distance movement, aid in viral replication and virion maturity, and promote insect host transmission and retention. For an in-depth discussion of the multifunctional activities of plant virus proteases, we refer the readers to a recent review [16]. In many cases, the diverse biological functions of viral proteases are directly related to their proteolytic activity. For example, the controlled cleavage of polyproteins encoding viral coat proteins, replication proteins or suppressors of silencing regulates virion maturation, viral RNA replication, and viral counter-defense responses. In addition, many animal and human virus proteases do not only cleave the viral polyproteins but also host proteins to facilitate the various steps of the virus infection cycle (translation, replication, suppression of host defense responses) [17,18]. In other cases, viral proteases have additional functions that are separate from their proteolytic activities. For example, different domains of the plant potyvirus HC-Pro protease have been implicated in proteolytic cleavage, suppression of antiviral RNA silencing, and aphid transmission [26]. Finally, the multifunctional activities of proteases can also overlap in their three-dimensional structure (3D structure), as shown for the dual protease-deubiquitinase of turnip yellow mosaic virus [27]. As will be described below, the multifunctional activities of viral proteases have influenced their evolution, in some cases resulting in the adoption of simplified folds for their proteolytic domains as compared to their cellular counterparts.

In this review, we discuss the diversity, evolution, and possible origin of various protease domains found in plant viruses. We also provide examples of possible exchange of protease domains among unrelated plant viruses. Please note that we did not attempt to list every protease signature motifs that were identified in virus genome annotations, rather we have focused the text on well-characterized proteases with known activities, specificities, and/or structures. Throughout the text, we have adopted the terminology of the MEROPS database (available at www.ebi.ac.uk/merops) for protease clans and families [28]. For virus taxonomy (genera, family, order), we use the 2018 taxonomy release of the International Committee for the Taxonomy of Viruses (available at https://talk.ictvonline.org/taxonomy/) [29].

## 2. Functional Protease Types

Proteases are classified in clans and families, which are based on their catalytic types (serine, threonine, cysteine, aspartic, glutamic, asparagine, and metallo), phylogenies, and molecular structures [28]. The catalytic mechanisms are best understood for the prevalent serine, cysteine, aspartic, and metalloproteases. Serine and cysteine proteases use the hydroxyl group (serine, threonine) or sulfhydryl group (cysteine) of the catalytic residue side chain as the catalytic nucleophile and a histidine as a base residue. Metallo and aspartic proteases require a water molecule as a nucleophile. Aspartic and glutamic proteases are considered acid proteases and normally function optimally at low pH. The vast majority of plant cellular proteases are cysteine, serine, aspartic, and metalloproteases, many of which have been implicated in plant defense responses [30,31].

Until recently, proteases that are encoded by plant (+)-strand RNA viruses were reported to belong to two clans: chymotrypsin-like cysteine or serine proteases (clan PA) and papain-like cysteine proteases (clan CA) [16,28]. In addition, plant reverse-transcribing viruses encode pepsin-like aspartic proteases (clan AA). As will be described below, the structural and functional characterizations of cysteine and serine proteases from various (+)-strand RNA viruses have led to new insights in their diversity and specialization. Many plant virus proteases have ancient origins, for example, the chymotrypsin-like cysteine or serine proteases of picorna-like viruses [4]. Although related in structures to their cellular counterparts, these viral proteases show little sequence identities other than a few conserved catalytic residues and they display more stringent cleavage site specificities [32,33,34]. In contrast, other plant virus proteases are likely to be more recent acquisitions, such as the newly described glutamic protease of strawberry mottle virus [35].

## 3. Chymotrypsin-Like Serine and Cysteine Proteases

**Figure 1:**
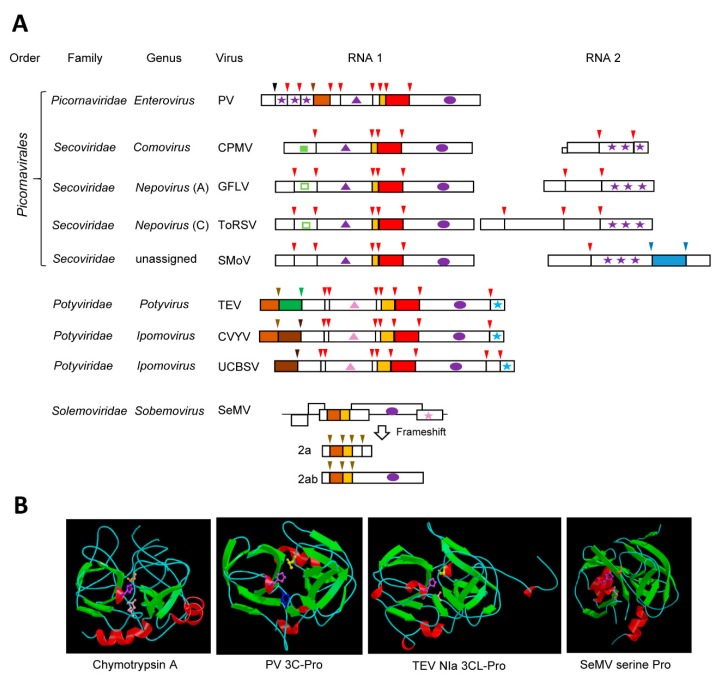
Chymotrypsin-like cysteine or serine proteases. (**A**) Genomic organization of representative viruses. Polyproteins are shown by boxes with cleavage sites indicated by vertical lines. Protease domains are shown in red for chymotrypsin-like 3C or 3CL cysteine proteases, brown for chymotrypsin-like serine proteases, green for papain-like cysteine proteases, and blue for glutamic proteases. The two shades of brown in the polyprotein of potyvirids represent P1 serine proteases of type A (light brown) or type B (dark brown). The same color code is used for arrows above each cleavage site indicating the protease responsible for the cleavage. A black arrow in the PV polyprotein indicates an autocatalytic maturation cleavage event of the capsid protein. Orange boxes indicate the VPg proteins. Purple ovals represent the conserved picorna-like RdRp domain. Stars indicate coat protein domains: purple for the three picorna-like type 1 jelly-roll domains (which can be divided into one, two, or three CPs, depending on the genera), pink for type 2 jelly-roll domains, and blue for CPs forming filamentous virions. Helicase domains are shown by the triangles: purple for superfamily 3 helicases and pink for superfamily 2 helicases. The protease co-factor motif of CPMV is shown by a small green square. This motif is present in nepoviruses (represented by the empty green square) but does not act as a co-factor. (**B**) Structure of representative proteases. Catalytic residues are represented as follows. Cellular protease: chymotrypsin A from *Bos taurus* (pdb:1CBW_ABC) His^57^ (purple), Asp^102^ (pink), Ser^195^ (orange), human virus protease: PV 3C-Pro (pdb: 1L1N_A): His^1603^ (purple), Glu^1634^ (blue), Cys^1710^ (yellow) and plant virus proteases: TEV NIa 3CL-Pro (pdb: 1LVM): His^2083^ (purple), Asp^2118^ (pink), Cys^2188^ (yellow), and SeMV serine Pro (pdb: 1ZYO): His^181^ (purple), Asp^216^ (pink), Ser^284^ (orange). Images of protease structures are reprinted with permission from the MEROPS database (www.ebi.ac.uk/merops). PV: poliovirus, CPMV: cowpea mosaic virus, GFLV: grapevine fanleaf virus, ToRSV: tomato ringspot virus, SMoV: strawberry mottle virus, TEV: tobacco etch virus, CVYV: cucumber vein yellowing virus, UCBSV: Ugandan cassava brown streak virus, SeMV: sesbania mosaic virus.

Many plant (+)-strand RNA virus proteases are cysteine or serine proteases that share structural homologies with the cellular chymotrypsin serine proteases. These chymotrypsin-like proteases are encoded by viruses of the “picorna-like supergroup”, which, although infecting a variety of eukaryotic hosts, are grouped together based on the common origin of their RdRps [1,4,36]. Notable chymotrypsin-like viral proteases include the archetype 3C cysteine proteases (3C-Pro) of animal and human viruses in the family *Picornaviridae* [36,37] and the related 3C-like proteases (3CL-Pros) of plant viruses in the families *Secoviridae* [38] and *Potyviridae* [39] (Figure 1A). Serine proteases of plant viruses in the family *Solemoviridae,* and in the genera *Polerovirus* and *Enamovirus* also adopt a chymotrypsin-like fold. Relationships between these viral proteases and the cellular chymotrypsin serine protease were identified more than 30 years ago based on sequence alignments and mutagenesis studies [40,41,42,43,44,45], and these were confirmed almost a decade later with the elucidation of the crystal structures of several 3C-Pros [46,47,48,49]. Viral chymotrypsin-like proteases are very diverse. This is likely due to their ancient origin. Indeed, phylogenetic relationships with bacterial serine membrane proteases led to the suggestion that a chymotrypsin-like protease was acquired from a bacterial endosymbiont by an ancestral picorna-like virus during eukaryogenesis [4].

The structure of chymotrypsin is characterized by a double-barrel fold, which is shared by the viral chymotrypsin-like proteases (Figure 1B). Chymotrypsin activity is dependent on a catalytic triad consisting of His^57^, Asp^102^, and the nucleophile Ser^195^, which are brought together in the 3D structure. The base His residue is highly conserved in all viral chymotrypsin-like proteases. The nucleophile Ser residue is conserved in the viral serine proteases, but it is replaced by Cys in the 3C- and 3CL-Pros [36,37]. The Asp residue is substituted by Glu in some viral proteases [36,50]. In contrast to the relaxed specificity of chymotrypsin, viral chymotrypsin-like proteases have stringent cleavage site specificities, which are determined by the shape and depth of their substrate-binding pocket (SBP) [36,37,51]. A conserved His in the S1 position of the SBP of most chymotrypsin-like viral proteases is responsible for the recognition of cleavage sites with a Gln (or Glu) at the P1 position. Other residues in the P6 to P2’ positions of the cleavage sites also contribute to the protease specificity. Finally, the folding of the polyprotein substrate determines the cleavage site accessibility to the protease [52,53].

### 3.1. The Archetype Picornavirus 3C-Pros

Proteolytic processing was shown to be a prerequisite for the formation of mature proteins of human and animal picornaviruses in the late 1960s [54]. The protease activity was assigned to the 3C protein ten years later [55,56,57]. In the case of the 3C-Pro of poliovirus (PV, species *Human enterovirus C*, genus *Enterovirus*), the catalytic triad consists of His^40^, Glu^71^, and Cys^147^. The spacing of the residues forming the catalytic triad is tighter than that of chymotrypsin, and this was attributed to deletions in exposed surface loops. The overall 3D structure of picornavirus 3C-Pros is otherwise similar to that of chymotrypsin [47,48] (Figure 1B). Although other proteases are encoded by picornaviruses, the 3C-Pro is responsible for the majority of the cleavage events in the viral polyproteins [36,58] (Figure 1A). The picornavirus 3C-Pros are active in the context of large polyproteins and cleave at several sites with different efficiencies. Primary cleavage occurs rapidly, allowing for the accumulation of intermediate polyproteins, which are then processed sequentially at secondary cleavage sites [18]. In addition to their essential role in viral polyprotein processing, the picornavirus 3C-Pros bind the viral RNA and enhance viral RNA replication [18], a property that is shared by the 3CL-Pros of the plant potyviruses [59] and possibly other plant picorna-like viruses. RNA binding is facilitated by conserved residues that are located on a surface opposite of the catalytic site in the 3D structure [47,48]. Picornavirus 3C-Pros also facilitate viral infection by cleaving various host proteins, notably translation and transcription factors, nuclear pore proteins, and proteins implicated in the innate immune response [17,18,19]. Whether similar host protein cleavage is also directed by the plant virus 3CL-Pros has so far not been investigated.

### 3.2. The 3CL-Pros of Viruses in the Family Secoviridae and Their Diverse Cleavage Site Specificities

The family *Secoviridae* is a large family of plant viruses that mostly infects horticultural crops [38]. Viruses in the family *Secoviridae* (secovirids) share capsid structures and signature replication proteins with picornaviruses and they are included in the order *Picornavirales* [22,23,38,60]. Secovirids can have monopartite or bipartite genome and they encode large polyproteins (Figure 1A). Until very recently, secovirids were only known to encode a single protease, the 3CL-Pro, which processes the viral polyproteins *in cis* and *in trans*. All secovirid 3CL-Pros share the conserved catalytic triad of the picornavirus 3C-Pros. Most have the signature replacement of the chymotrypsin nucleophile Ser by Cys, with the notable exception of the 3CL-Pro of blueberry latent spherical virus (genus *Nepovirus*), which has retained the Ser [61].

The cowpea mosaic virus (CPMV, genus *Comovirus*) 3CL-Pro, often referred to as the 24K protease, was the first secovirid 3CL-Pro identified [62]. Other well-characterized secovirid 3CL-Pros include those of grapevine fanleaf virus (GFLV) and tomato ringspot virus (ToRSV), two members of the genus *Nepovirus* [63,64] (Figure 1A). Comoviruses and nepoviruses have a bipartite genome. Each RNA encodes a single large polyprotein, with the protease domain being contained in the RNA1 polyprotein. Cleavage of comovirus and nepovirus polyproteins is sequential, leading to the accumulation of multiple intermediates and mature proteins [38,65,66]. The proteolytic activity of the comovirus and nepovirus 3CL-Pros is regulated by the presence of other viral protein domains. In the case of CPMV, a viral co-factor protein (the 32K protein, also encoded by RNA1) slows down the *cis*-cleavage of the RNA1 polyprotein but it enhances the *trans*-cleavage of the RNA2 polyprotein [67]. This allows the accumulation of several intermediate RNA1 polyproteins containing two or more protein domains. Although a corresponding protein domain is present in nepovirus RNA1 polyproteins, it does not impact the activity of nepovirus 3CL-Pros [68,69]. Rather, the activity of these proteases is influenced by the presence of the genome-linked viral protein (VPg) domain on VPg-Pro intermediate polyproteins. Cleavage at nepovirus VPg-Pro cleavage sites is inefficient, leading to the accumulation of the VPg-Pro or larger precursors, from which the mature Pro is slowly released [70,71]. The mature Pro of two nepoviruses (ToRSV and GFLV) is more active than the VPg-Pro in cleaving the RNA2 polyprotein *in trans* to release the coat proteins [71,72], adding a regulatory step to delay virus encapsidation.

The consensus sequence of CPMV cleavage sites is Ax(A,P)Q↓(S,G or M) and includes a strictly conserved Gln at the P1 position and preferred residues at the P1’, P2, and P4 positions with the down-arrow indicating the exact position of the cleavage. This consensus sequence is similar to that of the 3C-Pros of many picornaviruses, owing to the presence of the conserved His in the protease SBP [73]. Systematic mutagenesis of the ToRSV cleavage sites also showed a preference for *trans*-cleavage at sites conforming to the consensus sequence (C,V)Q↓(G,S) [74]. Interestingly, the specificities of the CPMV and ToRSV proteases were shown to be more relaxed for cleavage sites that were recognized *in cis* [53,74]. While many secovirid 3CL-Pros share a preference for (Q,E) ↓(S,G) cleavage sites, this is not universally observed in this family [38,65]. In fact, secovirid 3CL-Pros show surprising differences in their specificity. Some lack the conserved His in their SBP, which is replaced by Leu (nepoviruses of subgroup A and B, sequiviruses), Val (stocky prune virus), or Cys (sadwaviruses). These proteases recognize a variety of atypical cleavage sites with Arg, Lys, Gly, Cys, Thr, or Ala at the P1 position. Additionally, some proteases with the conserved His present in the SBP have relaxed specificities recognizing D/S, N/S, or even C/S cleavage sites [38,65]. Elucidation of the crystal structures of secovirid 3CL-Pros would be required to understand how their SBPs accommodate these unusual cleavage site specificities. The evolutionary constraints that drove these diverging protease specificities are not well understood. For example, although many nepoviruses share similar infection cycles and host ranges, they differ widely in their protease specificity [65]. If nepovirus proteases target similar host proteins, then they must have adapted to cleave these proteins at different sites. This question should be a fruitful area of research in the future.

### 3.3. The well-characterized potyvirus NIa proteases

The family *Potyviridae* is the largest and most economically important family of plant (+)-strand RNA viruses [39]. Although often referred to as “picorna-like” viruses because of the signature RdRps and 3CL-Pros that they encode, viruses in the family *Potyviridae* (potyvirids) differ from members of the order *Picornavirales* by their capsid structure and by the nature of the helicase and VPg [22,39] (Figure 1A). The 3CL-Pro of potyvirids is referred to as the NIa protease, an abbreviation for nuclear inclusion protein a. Although potyvirids encode other proteases (Figure 1A), the NIa protease is responsible for most of the viral polyprotein cleavage events and it can act *in cis* or *in trans*, depending on the cleavage site [39,51]. The NIa protease of tobacco etch virus (TEV, genus *Potyvirus*) is well characterized. It has a stringent specificity, recognizing cleavage sites with the consensus sequence ExxYxQ↓(S or G). The requirement for a Gln at P1 position and for small residues at the P1’ position is consistent with that of most other 3CL-Pros, while the preference for specific residues at the P3 and P6 position is more unique. The overall 3D structure of the TEV NIa protease was found to be similar to that of the 3C-Pro of human and animal picornaviruses [75] (Figure 1B). The specificity of the TEV NIa protease was confirmed by mutagenesis studies and later explained by the conformation of the SBP in the crystal structure [75,76,77]. The TEV NIa protease has been developed into a versatile biotechnology tool, facilitating the removal of fusion tags following affinity purification of recombinant proteins or protein complexes and allowing the simultaneous expression of multiple proteins in plant, mammalian, or bacterial cells [78,79,80,81,82,83,84,85].

Other well-characterized potyvirus NIa proteases include those of plum pox virus (PPV), tobacco vein mottling virus (TVMV), and turnip mosaic virus (TuMV) [86,87,88,89,90]. Like the TEV NIa protease, they recognize cleavage sites with a Gln in the P1 position and small residues at the P1’ position. However, they require a Val at the P4 position, and either a His (PPV, TuMV) or Phe (TVMV) at the P2 position of the cleavage sites. Structure comparisons revealed differences in the SBPs of the TEV and TVMV NIa proteases that explain their contrasting specificities [91]. Most other NIa proteases have similar specificities, with the notable exception of the sweet potato mild mottle virus (SPMMV, genus *Ipomovirus*) NIa protease that lacks the conserved His in the SBP (replaced by Asn) and recognizes cleavage sites with a His at the P1 position [51]. Experiments that were designed to alter protease specificities by exchanging fragments or modifying specific amino acids in the SBP resulted in overall poor protease activities, revealing that long-distance interactions contribute to the proper folding of these highly adapted proteases [90,92].

The *trans*-processing activity of the NIa protease is impacted by its maturation stage. Indeed, the VPg-Pro intermediate has been shown to be more active than the mature Pro [93,94]. The cleavage site between the VPg and protease domains is deliberately sub-optimal (with Glu instead of Gln at the P1 position), allowing the accumulation of VPg-Pro intermediate polyproteins in infected plants [95]. The VPg is naturally unstructured [94,96]. The N-terminal 22 amino acids of the VPg are essential to maintain the disordered state of the protein and to enhance the processing activity of the protease [94]. Given that the VPg is also known to interact with a large number of host proteins [97,98], it is possible that its presence in VPg-Pro intermediates facilitates the cleavage of host proteins. However, this has not yet been investigated.

### 3.4. The Sobemovirus Serine Protease

A putative serine protease domain with homology to cellular and viral chymotrypsin-like proteases was first identified in the genome of southern bean mosaic virus (genus *Sobemovirus*, family *Solemoviridae*) [42]. The serine protease domain is conserved in all members of the family *Solemoviridae*, which also includes the genus *Polemovirus* [99]. A closely related serine protease is also found in the genome of viruses in the genera *Enamovirus* and *Polerovirus*, which are currently classified in the family *Luteoviridae* but also share the Pro-VPg-RdRp replication module with the family *Solemoviridae* [99]. The serine proteases from this group of viruses show the characteristic catalytic triad (His^181^, Asp^216^, and Ser^284^ for sesbania mosaic virus, SeMV) [100,101,102]. Cleavage sites with the consensus sequence E↓(S,T) are recognized by the polerovirus and sobemovirus serine proteases [100,102,103], which is similar to the cleavage site specificity of most 3CL-Pros. Accordingly, the conserved His of the 3CL-Pro SBPs is also present in this group of serine proteases. The structure of the SeMV protease was solved, which confirmed a double-barrel domain fold that is typical of chymotrypsin-like proteases and the role of the conserved His in the SBP [104] (Figure 1B).

The regulation of SeMV proteolytic cleavage is interesting. Presence of the intrinsically unfolded VPg domain is strictly required for the SeMV protease activity [105]. In contrast to other picorna-like viruses, the VPg domain is C-terminal to the serine protease domain in the polyprotein (Figure 1A). Exposed aromatic residues in the protease domain interact with a tryptophane in the VPg domain to facilitate the interaction and the activation of the protease [104,106]. SeMV encodes two polyproteins 2a and 2ab, which share identical N-terminal domains (including the Pro domain and several cleavage sites) but differ in their C-terminal domains [99]. This is due to an inefficient frameshift in the RdRp domain, which allows the formation of the longer 2ab polyprotein (Figure 1A). Interestingly, the processing efficiency at cleavage sites that is shared by the two polyproteins differ considerably between polyprotein 2a or 2ab, suggesting that the presentation of the cleavage sites to the protease is influenced by the conformation of the polyproteins [102]. These results highlight the unique mechanisms regulating the activity of the SeMV serine protease.

### 3.5. The Compact and Diverse Potyvirid P1 Serine Proteases and Their Intricate Regulatory Mechanisms

The P1 protein is a minor protease that is present in some but not all members of the family *Potyviridae* [39,51] (Figure 1A). Located at the N-terminus of the polyprotein, it directs a single *cis*-cleavage to release itself from the polyprotein [107]. The protease activity was mapped to the C-terminal region of the P1 protein domain. His^214^, Asp^223^, and Ser^256^ were identified as catalytic residues for the TEV P1 protease [108]. Although these residues are typical of serine protease catalytic triads, they are more closely spaced than any other viral cysteine or serine chymotrypsin-like proteases. Analysis of other potyvirid P1 proteases revealed similar tight arrangements of the putative catalytic triad, with only seven to nine residues separating the His and Asp (or Glu) residues [50]. The cleavage site consensus sequence is (V,I,L,M)xx(Y,F)↓S, which is conserved for all viruses in the family *Potyviridae* that have a P1 protease [51]. The protease structure has not yet been determined and the conformations of the catalytic site or of the SBP are not well understood.

Potyvirus P1 proteases are diverse, varying in their size and amino acid sequence [50]. Some viruses even have two copies of the P1 proteases, for example, cucumber vein yellowing virus (CVYV, genus *Ipomovirus*) [50,109,110] (Figure 1A). Most of the P1 diversity was attributed to the variable N-terminal region, which has been implicated in host specificity and virus virulence [111,112,113]. Complex schemes of gene duplication and recombination have been proposed to explain the evolution and diversity of P1 proteases [50]. P1 proteases have been divided into two main types based on sequences and regulatory mechanisms [50,110,114]. Type A includes most potyvirus P1 proteases and the first of the two CVYV P1 proteases (termed P1a). The second CVYV P1 protease (P1b) and the Ugandan cassava brown streak virus P1 protease are examples of type B. Mechanistically, the two types differ in that type B proteases are fully functional, while type A proteases depend on a plant factor for their activity [110,114]. Indeed, it was noted early that, while the activity of the full-length TEV P1 protease is easily detected in wheat germ extracts or upon expression in plants, the protease is not active in rabbit reticulocyte extracts unless a heat-labile plant factor present in wheat germ extracts is supplied to the reaction [108]. Later work demonstrated that the N-terminal region of P1 is antagonistic to the protease activity. This antagonism is relieved by binding to an as-yet unidentified plant factor in a host dependent manner or by the deletion of the N-terminal region [110,114]. The antagonistic regulation of type A P1 proteases has been linked to host range and viral virulence [112,113,114,115]. Strikingly, introducing deletions of the P1 N-terminal region in PPV infectious clones resulted in increased virulence and host range [112,114]. Efficient cleavage by the P1 and P1a proteases is necessary to activate the silencing suppression activity of downstream protein domains (HC-Pro and P1b, respectively), thereby counteracting plant antiviral defenses. This sophisticated protease regulatory mechanism demonstrates a remarkable level of adaptation of potyvirids to their hosts.

## 4. The Diverse “Papain-Like” Cysteine Proteases

**Figure 2:**
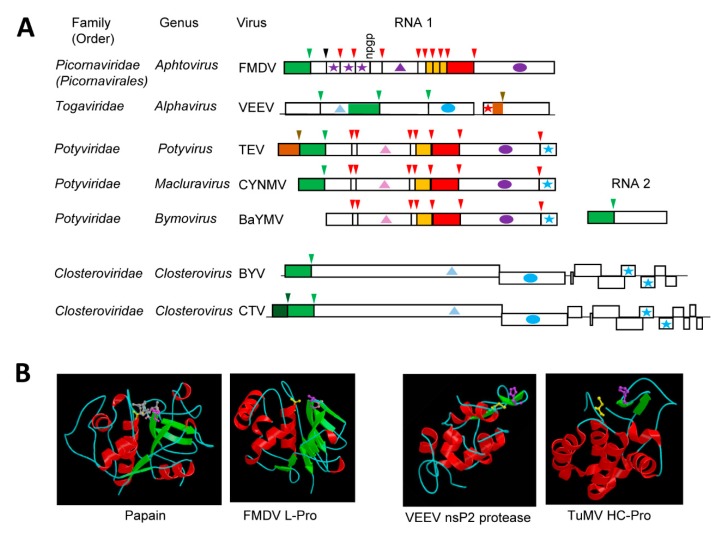
Papain-like cysteine proteases. (**A**) Genomic organization and proteolytic cleavages of representative viruses. Polyproteins are shown by boxes with cleavage sites indicated by vertical lines. Protease domains are shown in green for papain-like cysteine proteases, red for chymotrypsin-like 3CL cysteine proteases and brown for chymotrypsin-like serine proteases. The same color code is used for arrows above each cleavage site indicating the protease responsible for the cleavage. A black arrow in the foot-and-mouth disease virus (FMDV) polyprotein indicates an autocatalytic maturation cleavage event of the capsid protein. The “ngpg” sequence represent the 2A translational stop-go sequence of FMDV. Orange boxes indicate the VPg proteins. Ovals represent RdRp domains: purple for picorna-like RdRp and blue for alpha-like RdRp. Stars indicate the coat protein domains: purple for picorna-like type 1 jelly-roll domains icosahedral coat protein, red for unrelated icosahedral CP of togaviruses, blue for filamentous coat proteins. Helicase domains are shown by the triangles: light blue, pink and purple for helicase superfamilies 1, 2, and 3, respectively. Please note that cleavage sites processed only by viral proteases are shown. The VEEV cleavage sites processed by the cellular furin protease are not shown. (**B**) Structure of representative proteases. Catalytic residues are represented, as follows; Cellular protease: papain from *Carica papaya* (pdb: 1PE6, in complex with E-64 inhibitor, which is shown in grey): Cys^158^ (yellow), His^292^ (purple), Asn^308^ (pink), animal virus proteases: FMDV L-Pro (pdb: 1QMY_A: mutant protease with Cys^51^ mutated to Ala), Cys^51^ (yellow, mut. to Ala), His^148^ (purple), Asp^163^ (pink), and VEEV nsP2 protease (pdb: 2HWK, only the protease catalytic domain is shown): Cys^477^ (yellow), His^546^ (purple), and plant virus protease: TuMV HC-Pro (pdb: 3RNV, protease catalytic domain): Cys^706^ (yellow), His^779^ (purple). Images of protein structures are reprinted with permission from the MEROPS database (www.ebi.ac.uk/merops). FMDV: foot-and-mouth disease virus, VEEV: Venezuelan equine encephalitis virus, TEV: tobacco etch virus, CYNMV: Chinese yam necrotic mosaic virus, BaYMV: barley yellow mosaic virus, BYV: beet yellows virus, CTV: citrus tristeza virus, TuMV: turnip mosaic virus.

The cellular papain cysteine protease is a globular protein with two interacting domains: an N-terminal helical domain (termed R domain) that includes the nucleophile Cys^158^ residue and a C-terminal domain mostly composed of β–sheets (termed L domain) that encompasses the base His^292^ residue, 134 amino acid downstream of the Cys^158^ residue (Figure 2B) [116]. The two catalytic residues are brought in close proximity in the 3D structure. A third residue (Asn^175^) forms a hydrogen bond with Cys^158^. However, mutagenesis studies did not confirm a strict requirement of Asn^175^ for proteolytic activity [117], and the catalytic site is generally considered to function as a dyad. Papain-like proteases represent a large and diverse group of proteases (clan CA) [118].

Possible relationships between papain and some (+)-strand RNA virus proteases were identified in the early 1990s and they were based on sequence alignments that confirmed the presence of conserved cysteine and histidine residues [41,119]. Elucidation of the structure of the leader protease (L-Pro) of foot-and-mouth disease virus (FMDV, genus *Aphtovirus*, family *Picornaviridae*) revealed a papain-like fold with Cys^51^, His^148^, and Asp^163^ (replacing the Asn of papain) forming the catalytic cleft [120] (Figure 2B). The FMDV L-Pro is an accessory protease. It is not conserved in all members of the family and it processes the polyprotein *in cis* at a single KLK↓GAG cleavage site to release itself (Figure 2A). In contrast, the nsP2 cysteine protease of Venezuelan equine encephalitis virus (VEEV, genus *Alphavirus*, family *Togaviridae*) is the main protease and it cleaves the nonstructural polyprotein at four sites (Figure 2A). The structure of the VEEV cysteine protease revealed major divergence from the papain fold [121] (Figure 2B). The C-terminal region of the nsP2 protein (which contains the protease domain) includes the catalytic protease domain and a C-terminal methyl-transferase like domain. The protease domain differs from a classical papain fold, in that it is mainly helical. The N-terminal region shares some similarities with the papain helical R domain, while a simplified L domain contains only two short β-sheets. The two catalytic residues Cys^477^ and His^546^ are more closely spaced than in papain, with only 69 residues separating them. However, they are brought together in the catalytic site to adopt a conformation that is similar to that of the papain fold. An equivalent to the papain Asn^175^ residue was not found.

Not surprisingly, several plant (+)-strand RNA viruses also encode cysteine proteases. A putative papain-like protease was initially identified in the polyprotein of potyviruses [119]. Since then, other plant viruses were predicted to encode cysteine proteases, which have been referred to as papain-like proteases. However, as will be discussed below, the structure of characterized plant virus cysteine proteases differ from that of papain in many ways and reflect the unique adaptations and/or diverse origins of these enzymes.

### 4.1. The Multifunctional Potyvirid HC-Pro Protease with a Minimalistic Papain-Like Fold

Like the P1 protease, the helper component protease (HC-Pro) of potyvirids is a minor protease that cleaves the polyprotein *in cis* at a single site [122,123] (Figure 2A). Also similar to the P1 protease, not all viruses in the family *Potyviridae* encode an HC-Pro domain [26,39]. The cleavage site consensus sequence, YxG↓G, is strictly conserved amongst the monopartitite potyvirids [51]. Viruses in the genus *Bymovirus* have a bipartite genome and also encode a related cysteine protease, which is termed P2-1. The P2-1 protease is located in the N-terminal region of the RNA2 polyprotein and it cleaves at a single related cleavage site with the consensus sequence of (Y,F, G)xG↓(A, N, S) [51] (Figure 2A).

The multi-functional HC-Pro plays roles in vector transmission and in the suppression of antiviral plant defense responses, notably RNA silencing [26]. HC-Pro is a multi-domain protein, with the protease catalytic region present in the C-terminus of the protein [124]. While the presence of the protease domain is conserved in all potyvirids that encode the HC-Pro protein, other domains that are involved in RNA binding, silencing suppression, and vector transmission vary widely within the family [26]. Even though HC-Pro was dubbed a papain-like protease, it was noted early on that the spacing between the catalytic residues (Cys^706^ and His^779^ for TuMV) is shorter than that observed in papain or in the leader proteases of animal and human picornaviruses [119]. The crystal structure of the C-terminal protease domain of the TuMV HC-Pro protein confirmed a simplified papain-like fold with some similarities to that of the VEEV nsP2 protein, including the presence of two short β-sheets forming the corresponding papain R domain [125] (Figure 2B). As with the animal alphavirus nsP2 protein, the two catalytic residues of the plant potyvirus HC-Pro are brought together in a papain-like topology. Interestingly, the C-terminus of HC-Pro was found locked into the active site cleft, preventing further cleavage and providing an explanation for the strict *cis*-cleavage mechanism [125]. The structure of other regions of the protein has not been determined with confidence, but phylogenetic analyses of separate domains coupled with structure modelling suggested that the multi-functionality of the protein drove the co-evolution of its domains [126]. It is possible that these overlapping selection pressures contributed to the evolution of a minimalistic papain-like fold for the protease domain.

### 4.2. The single or Tandem Closterovirus Leader Proteases

Viruses in the family *Closteroviridae* have the longest (+)-strand RNA genome (between 15 and 20 kb) amongst plant viruses [25,127]. The replication proteins are expressed as a large polyprotein that is cleaved at a single site by the leader protease (L-Pro), which is located at the N-terminus of the polyprotein (Figure 2A). The protease of beet yellows virus (BYV, genus *Closterovirus*) was first characterized in 1994 [128]. It was shown to have limited sequence similarities with other viral papain-like proteases, notably the potyvirus HC-Pro, and to require catalytic residues Cys^509^ and His^569^ to cleave at the single G↓G cleavage site [128,129]. Similar to HC-Pro, L-Pro is a multifunctional and multi-domain protein that has been implicated in the regulation of virus accumulation, long-distance transport, and host adaptation [130,131]. Also similar to HC-Pro, the protease catalytic domain is contained in the highly conserved C-terminal region of L-Pro, while the more variable N-terminal region orchestrates the other biological activities. The structure of the protease has not been determined and it not known whether it adopts a simplified papain-like fold that is similar to that of the plant potyvirus HC-Pro and the animal alphavirus nsP2 protease.

It is interesting to note that some members of the family *Closteroviridae* encode two L-Pros, notably citrus tristeza virus (CTV) and grapevine leafroll-associated virus 2 (GLRaV-2). The tandem proteases probably arose by domain duplication [132,133,134]. In model herbaceous hosts, the first L-Pro copy of either GLRaV-2 or CTV is strictly required for achieving infection, while the second copy plays accessory roles [132,134]. In contrast, both copies are strictly required for infection in the natural perennial hosts (grapevine and citrus, for GLRaV-2 and CTV, respectively). Cleavage after the first GLRaV-2 L-Pro is dispensable, as long as cleavage occurred at the second site to release the viral replication proteins [132]. It has been suggested that the acquisition of a second protease domain may assist in host range expansion [132,134].

### 4.3. The Tymovirus Cysteine Protease with a Compact Ovarian-Tumor (OTU) Domain-Like Fold Driven by Its Dual Function as a Protease and Deubiquitinase

A new superfamily of cysteine proteases was proposed in 2000 that shares the catalytic Cys and His residues with papain, but has sequence relationships closer to the Ovarian Tumor Domain (OTU) proteins than to papain [135]. Similar to papain, the OTU-like proteins include a conserved Asp or Asn residue to form a catalytic triad. This OTU-like superfamily includes sequences from several viruses that are currently classified in the order *Tymovirales, Nidovirales* and *Bunyavirales* (an order encompassing a diverse group of negative-strand RNA viruses). It is now well-established that many OTU-like cysteine proteases have deubiquitinase (DUB) activity and cleave lysine-bound ubiquitin units at GG↓K cleavage sites [136].

**Figure 3:**
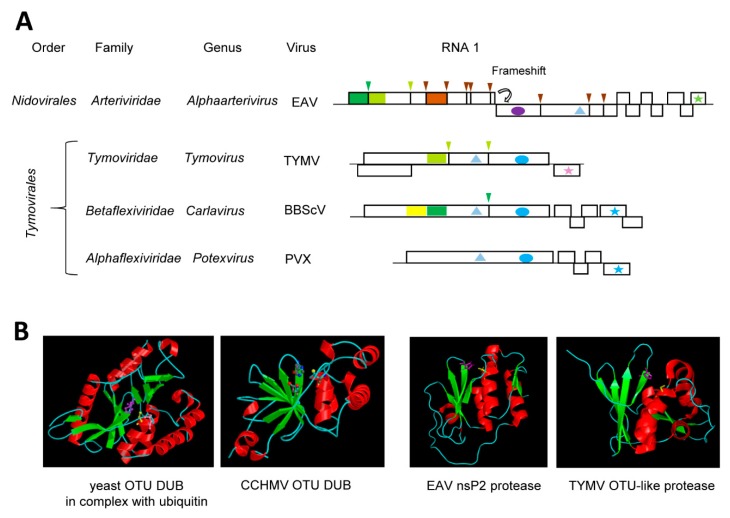
Ovarian Tumor Domain (OTU)-like cysteine proteases. (**A**) Genomic organization and proteolytic cleavages of representative (+)-strand RNA viruses. Polyproteins are shown by boxes with cleavage sites indicated by vertical lines. Protease domains are shown in dark green for papain-like cysteine proteases, lime green for OTU-like cysteine proteases and brown for chymotrypsin-like serine proteases. The same color code is used for arrows above each cleavage site indicating the protease responsible for the cleavage. The yellow square represents an OTU-like domain that does not orchestrate viral polyprotein cleavage. Ovals represent RdRp domains: purple for picorna-like RdRp and blue for alpha-like RdRp. Stars indicate the coat protein domains: green for the arteriviridae nucleocapsid, pink for type 2 jelly-roll domains and blue for filamentous coat proteins. Helicase domains are shown by the triangles: light blue for superfamily 1. (**B**) Structure of representative deubiquitinases (DUB) and/or proteases. Catalytic residues are represented as follows: Yeast OTU DUB (pdb: 3C0R, in complex with ubiquitin, only one monomer of the trimer is shown): Asp^177^ (pink), Cys^120^ (yellow), and His^222^ (purple). Animal negative-strand RNA virus DUB: CCHMV OTU DUB (pdb: 3PT2_A): Cys^40^ (yellow), His^151^ (purple), and Asp^153^ (pink). (Image of the yeast and CCHMV OTU DUB structures are reprinted with permission from the MEROPS database (www.ebi.ac.uk/merops)). Animal (+)-strand RNA virus protease-DUB: EAV nsP2 protease (pdb: 4IUM) Cys^270^ (yellow) and His^332^ (purple) and plant (+)-strand RNA virus protease-DUB: TYMV OTU-like protease (pdb: 4A5U): Cys^783^ (yellow) and His^869^ (purple). Image for the EAV and TYMV protease structure were generated using PyMol. EAV: equine arteritis virus, TYMV: turnip yellow mosaic virus, BBScV: blueberry scorch virus, PVX: potato virus X, CCHMV: Crimean-Congo hemorrhagic fever virus.

The cysteine protease of turnip yellow mosaic virus (TYMV, genus *Tymovirus*, family *Tymoviridae*, order *Tymovirales*) was initially referred to as papain-like, following the identification of the catalytic Cys^783^ and His^869^ residues in the mid-1990s [137,138]. However, it was noted early on that the protease differs from papain in many aspects, notably in the sequence of residues surrounding the catalytic residues [138]. For example, Cys^783^ was followed by Leu rather than by the aromatic residue normally found in papain-like proteases. Determination of the TYMV protease structure confirmed a fold with more similarities to the yeast OTU DUB than to papain [139] (Figure 3B). The TYMV protease does not only process the polyprotein at two sites (S↓Q and A↓T, suggesting a relatively relaxed specificity) [140] (Figure 3A), it also functions as a DUB to regulate the ubiquitination status and stability of the viral RdRp [141]. The structures of the TYMV OTU-like Pro-DUB, the OTU-like nsp2 Pro-DUB of equine arteritis virus (EAV, genus *Alphaarterivirus*, family *Arteriviridae*, order *Nidovirales*) and the OTU-DUB of Crimean-Congo hemorrhagic fever virus (CCHMV, a negative-strand RNA virus from the genus *Orthonairovirus*, family *Nairoviridae*, order *Bunyavirales*) are strikingly similar, although they also display interesting differences [136,139,142,143,144,145] (Figure 3B). In contrast to the TYMV and EAV proteases, the CCHMV DUB does not have proteolytic activity on viral proteins. The DUB activity of the TYMV OTU-like protease is very specific, preferring a subset of ubiquitinated substrates, notably the TYMV RdRp [141]. The TYMV Pro-DUB differs from related viral or cellular OTU-like DUBs in that it is more compact and that it also lacks the third catalytic Asp (or Asn) residue [136,139]. The TYMV protease has developed a clever mechanism to regulate its dual activities through reversible conformation changes [27]. The catalytic site can adopt open or closed conformations that favor the protease or DUB activities, respectively. This is regulated by a mobile loop located near the catalytic site that can form a rigid flap against the catalytic cleft. Mutations that affect the mobility of the loop and prevent the close conformation resulted in a loss of DUB activity while conserving the protease function [27]. Thus, the detailed functional and structural characterization of the TYMV protease has provided a fine example of the strong selection pressures at work to regulate the multiple activities of viral proteases.

The order *Tymovirales* encompasses a large group of viruses related by common signatures of their replication enzymes but are otherwise quite diverse, for example, having different capsid structures and/or different movement protein modules [24]. The acquisition and evolution of protease and DUB domains to regulate the processing and stability of the replication proteins is also complex. Although members of the family *Alphaflexiviridae* lack a protease domain, most members of the families *Betaflexiviridae* encode either a single cysteine protease or tandem OTU-like and papain-like motifs (Figure 3A). Indeed, the presence of duplicated putative Cys-His dyads was already noted in the polyprotein of blueberry scorch virus (BBScV, genus *Carlavirus*, family *Betaflexiviridae*) in the early 1990s with only the C-terminal dyad implicated in polyprotein cleavage [146]. The N-terminal dyad is consistent with an OTU-like motif and may be specialized in DUB activity although this will need to be confirmed experimentally [24] (Figure 3A). It has been hypothesized that the dual Pro-DUB function of the single TYMV protease may have been derived from an original OTU-like and papain-like dyad tandem, although alternative evolution scenarios are also possible [139]. Functional and structural characterization of additional proteases from the order *Tymovirales* will be necessary to gain a better understanding of their evolution history.

## 5. The Novel Glutamic Protease of Strawberry Mottle Virus (Family *Secoviridae*)

**Figure 4:**
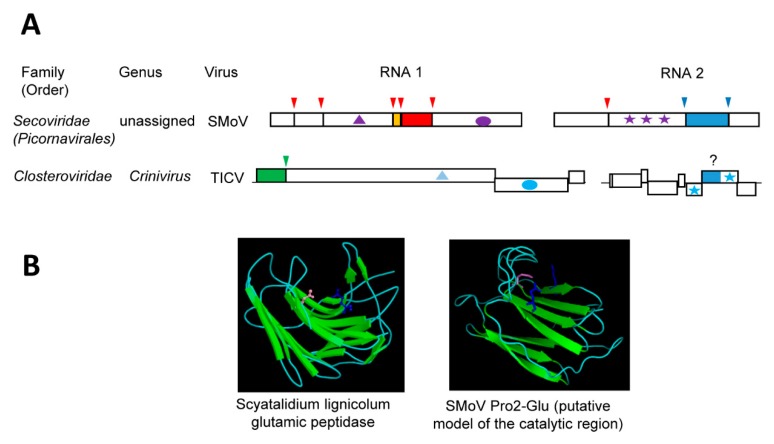
Confirmed and putative glutamic proteases. (**A**) Genomic organization and proteolytic cleavages of representative viruses. Polyproteins are shown by boxes with cleavage sites indicated by vertical lines. Protease domains are shown in green for papain-like cysteine proteases, red for chymotrypsin-like 3CL cysteine proteases, and blue for confirmed (SMoV) or putative (TICV) glutamic protease domain. The same color code is used for arrows above each cleavage site indicating the protease responsible for the cleavage. The orange box indicates the VPg protein. Ovals represent RdRp domains: purple for picorna-like RdRp and blue for alpha-like RdRp. Stars indicate the coat protein domains: purple for picorna-like type 1 jelly-roll domains icosahedral coat protein and blue for filamentous coat proteins. Helicase domains are shown by the triangles: light blue and purple for helicase superfamilies 1 and 3, respectively. (**B**) Structure of representative proteases. Catalytic residues are represented as follows: Fungal protease: Scyatalidium lignicolum glutamic peptidase (pdb: 1S2B): Gln^107^ (pink) and Glu^190^ (blue) (Image reprinted with permission from the MEROPS database (www.ebi.ac.uk/merops)) and plant virus protease: SMoV Pro2-Glu putative model of the catalytic region, generated using Phyre2 [35]: Glu^1192^ and Glu^1274^ (blue) and Gln^1180^ (pink). Image of the structure model was generated using PyMol. SMoV: strawberry mottle virus, TICV: tomato infectious chlorosis virus.

Until recently, members of the family *Secoviridae* (order *Picornavirales*) were only known to encode a single main protease (the 3CL-Pro, Section 3.2) to process the polyproteins at multiple sites [38]. However, strawberry mottle virus (SMoV, a bipartite member of the family, currently unassigned to a specific genus) was shown to encode a second protease of a novel type, which strictly requires two glutamic acid residues for its activity [35]. We refer to this novel protease as Pro2-Glu. While the RNA1-encoded 3CL-Pro cleaves the RNA1 polyprotein at five sites and the RNA2 polyprotein at one site, the RNA2-encoded Pro2-Glu cleaves the RNA2 polyprotein at two sites [35,147] (Figure 4A). The cleavage site consensus sequence of Pro2-Glu is P↓xFP. Cleavage by Pro2-Glu delineates two protein domains downstream of the CP domain, which is uncharacteristic for the family (Figure 1A). The first of these two domains contains the protease activity. Among secovirids, signature sequences for the Pro2-Glu domain were detected in only black raspberry necrosis virus and lettuce secovirus 1 (a putative new member of the family) [35]. The essential Glu^1192^ and Glu^1274^ residues are found in the conserved motifs M(F,Y)E (L,F,V)IWRF and GWEYQ, respectively. Mutation of two other conserved residues (Gln^1180^ and Gln^1322^) reduced the protease activity.

Homology-based modeling of the SMoV Pro2-Glu catalytic region implied a beta-sandwich structure that is typical of a concanavalin A-like lectin/glucanase fold [35] (Figure 4B). Although placed on different β-sheets, residues Glu^1192^, Glu^1274^, and Gln^1180^ are brought in close proximity in this model. Concanavalin A-like lectin/glucanases are a superfamily of proteins that share very low sequence identity and are found in a wide range of species, including microorganisms, insects, plants, and animals [148]. Most members of the superfamily have carbohydrate-binding activities and they influence an array of complex biological processes. Of interest, a group of bacterial and fungal glutamic proteases, which are collectively referred to as eqolisins (family G1, clan GA), also adopt the concanavalin A-like lectin/glucanase fold. The first described eqolisins was isolated from the plant pathogenic fungi *Scytalidium lignicolum* [149]. Structural and functional studies of eqolisins revealed a catalytic dyad consisting of glutamic acid (Glu^136^) and glutamine (Gln^53^) residues that are arranged on opposing beta-sheets [149,150,151,152,153]. Although the model of the SMoV Pro2-Glu protease catalytic domain shows some structural similarities with eqolisins, their amino acid sequence, the spacing between the catalytic residues, and the arrangements of the β-sheets differ significantly [35]. High-resolution structure analysis will be required to confirm the structure of the SMoV Pro2-Glu domain and its relationship with eqolisins or with other proteins with the concanavalin A-like lectin/glucanase fold. To date, there are no other viral proteases reported to adopt a similar fold. Whether the SMoV Pro2-Glu evolved from a divergent protease domain acquired from a plant pathogenic fungus or whether the protease catalytic activity developed from a host lectin protein (as suggested for eqolisins), is not clear. Either way, the unique proposed concanavalin A-like lectin/glucanases fold of Pro2-Glu, combined with the absence of this domain in most members of the family *Secoviridae,* suggest that it was relatively recently acquired.

A putative Pro2-Glu domain was detected in the minor coat protein (CPm) of several criniviruses and velariviruses (family *Closteroviridae*) [35]. Sequence motifs around the SMoV Pro2-Glu catalytic glutamic acid and glutamine residues were highly conserved in the CPms. Similar β-sheets structures were also predicted around the conserved sequences, suggesting a possible concanavalin A-like lectin/glucanases fold. Members of the family *Closteroviridae* incorporate the CPm in the short tail of the virion filamentous particle and the CPm is required for virus transmission by its aphid vector [25,154,155,156]. The CPm functions by binding to chitin sugar moieties on the cuticular surface of the aphid cibarium, thus anchoring citrus tristeza virions [157]. Competitive assays using monosaccharides or lectins reduced virion binding. Similar results were reported for the CPm of lettuce infectious yellows virus and its role in whitefly transmission [158]. These observations raise several interesting questions: (1) Does the SMoV Pro2-Glu domain have a similar lectin activity? If so, is it also involved in the transmission of SMoV by its aphid vector and does the protease activity contributes to regulating vector transmission? (2) Do the crinivirus and velarivirus CPms have glutamic protease activity? If so, what is the biological significance of this activity in vector transmission or in other aspects of the virus infection cycle?

## 6. Aspartic Proteases Encoded by Reverse-Transcribing Viruses and by a Plant Negative-Strand RNA Virus, but Not (Yet?) by (+)-Strand RNA Viruses

Reverse-transcribing viruses belonging to the order *Ortervirales* encode aspartic proteases that are related to the cellular pepsin and they are often referred to as retropepsin [159]. Retroviruses are restricted to vertebrate hosts and they are estimated to be the oldest known viral group [160]. The aspartic protease of human immunodeficiency virus 1 (HIV-1) is the best characterized and it has been shown to be essential for virion maturation [161]. The HIV-1 protease activity requires the formation of a homodimer, whereby each monomer confers an aspartate residue to form the active site (Asp^25^ and Asp^25^’) [162,163]. The family *Caulimoviridae* (order *Ortervirales*) is a family of plant-infecting pararetroviruses. Pararetroviruses share genomic organization, replication strategy, and phylogenetic relatedness with ancestral retroviruses but lack an integrase gene and package DNA instead of RNA into virions [159]. Cauliflower mosaic virus (CaMV, genus *Caulimovirus*) was the first pararetrovirus identified to encode an aspartic protease, which has also been implicated in virion maturation and regulation of the viral coat protein nuclear targeting [164,165,166]. Although the tertiary structure of caulimovirus proteases has not been determined, catalytic Asp residues that are found in the conserved D(T/S)G motif are assumed to adopt a homodimer configuration similar to that of retroviral proteases.

Recent findings revealed that aspartic proteases are not restricted to members of the order *Ortervirales*. Indeed, a retropepsin-like aspartic protease domain with a conserved DTG sequence was found to be encoded by a plant negative-strand RNA virus [167]. Citrus psorosis ophiovirus (CPsV, genus *Ophiovirus*, family *Aspiviridae*) is a tripartite virus that contains a protease domain within its movement protein. Mutation of the catalytic Asp residue prevented not only the maturation cleavage of the MP, but also the formation of tubular structures that are necessary for cell-to-cell movement of the virus. Whether the CPsV 20K protease was acquired from a plant reverse-transcribing element or from a cellular protease by parallel evolution is not known. To date, aspartic protease domains have not been identified with certainty in association with positive-strand RNA viruses. Although an early study identified a possible retropepsin-like DSG motif in the polyprotein of a closterovirus (BYV) [128], a proteolytic activity that is associated with this motif has not been confirmed experimentally. However, given the recent identification of a glutamic protease in a plant (+)-strand RNA virus [35], the possibility that they could also encode aspartic proteases cannot be excluded.

## 7. Conclusions

Detailed functional and structural analyses of viral proteases have provided new insights into the recurrent acquisition of proteolytic enzymes by viruses and into the adaptation of these protease domains to accommodate and facilitate virus infection cycles. The origin of some viral proteases are ancient, as exemplified by the main chymotrypsin-like serine and cysteine proteases of animal, plant, and lower eukaryotes picorna-like viruses that have limited sequence identities but share conserved catalytic residues and a similar general fold. As detailed in Section 3, this long history has driven diverse and intricate adaptations to facilitate and regulate the multi-functional activities of these viral proteases within the constraints of viral RNA genome size limitations. When compared to the cellular chymotrypsin, viral proteases have developed more stringent cleavage site specificities, which vary from one virus to another and are determined by the structure of their SBPs. Although specificity determinants are relatively well understood for the 3C-Pros of animal picornaviruses and 3CL-Pros of plant potyviruses and sobemoviruses, further work is required to understand the determinants and biological relevance of the divergent specificities of the 3CL-Pros of many plant viruses in the family *Secoviridae*. Understanding these protease specificities is critical not only for accurate prediction of viral polyprotein cleavage sites in emerging viruses, but also to identify host protein targets of viral proteases, an area that remains unexplored for plant viruses.

While the large family of chymotrypsin-like viral proteases share a common and ancient origin, other viral proteases have more complex evolutionary histories, which is reflected by the sequential acquisition of diverse protein domains, duplication and/or deletion of protein domains, and in some cases adaptation of non-proteolytic enzymes to attain protease activities (Section 4 and Section 5). It is clear that the evolution of these diverse proteases has been influenced by their multi-functional activities. Some viral proteases adopt multi-domain structures, where the protease activity is restricted to a small domain that is linked to other functional domains by flexible loops. These small protease domains often adopt a simplified fold when compared to their cellular counterparts, as exemplified by the minimalistic papain-like domains of the plant potyvirus HC-Pro or animal arterivirus nsP2 proteases (Section 4 and Section 4.1). Other viral proteases have adopted compact but flexible structures to achieve and regulate dual activities, for example the tymovirus OTU-like protease/deubiquitinase (Section 4.3). The catalytic mechanisms, biological functions and structures of many viral proteases remain to be examined. These include cysteine proteases from plant viruses that have been annotated as papain-like or OTU-like, such as the closterovirus leader proteases (Section 4.2) and the putative proteases of benyviruses [168] and cileviruses [169].

The recent discovery of an atypical glutamic protease encoded by a plant picorna-like virus (Section 5) implies that we have not yet fully explored the diversity of plant (+)-strand virus proteases. The SMoV glutamic protease is predicted to adopt a concanavalin A-like lectin/glucanase fold, suggesting that it has other as of yet unexplored activities. Structural studies will be required to confirm the predicted fold and to understand the catalytic mechanism of this novel viral protease. The discovery of an accessory glutamic protease encoded by a secovirid was unexpected, as other viruses in the family are not known to encode accessory proteases in addition to the main 3C-like protease. The SMoV glutamic protease presents little sequence identity with other proteins in available databases, and the prediction of its proteolytic activity would have been difficult without functional analysis. There are many other viral protein domains of “unknown functions” in sequence annotations, some of which may represent novel protease activities. Thus, it is likely that the repertoire of proteases encoded by plant (+)-strand RNA viruses will continue to expand in the years to come.
